# Easily detectable cytomorphological features to evaluate during ROSE for rapid lung cancer diagnosis: from cytology to histology

**DOI:** 10.18632/oncotarget.13204

**Published:** 2016-11-08

**Authors:** Sara Ravaioli, Sara Bravaccini, Maria Maddalena Tumedei, Flavio Pironi, Piero Candoli, Maurizio Puccetti

**Affiliations:** ^1^ Biosciences Laboratory, Istituto Scientifico Romagnolo per lo Studio e la Cura dei Tumori (IRST) IRCCS, Meldola, Italy; ^2^ Pathology Unit, Santa Maria delle Croci Hospital, Ravenna, Italy; ^3^ Pneumology Unit, Santa Maria delle Croci Hospital, Ravenna, Italy

**Keywords:** lung cancer, ROSE, histotype, cytomorphological features

## Abstract

In lung cancer patients, the only available diagnostic material often comes from biopsy or from cytological samples obtained by fine needle aspiration (FNA). There is a lack of easily detectable cytomorphological features for rapid on-site evaluation (ROSE) to orient lung cancer diagnosis towards a specific tumor histotype. We studied the cytological features evaluated on site to define tumor histotype and to establish the number of specimens to be taken. Cytological specimens from 273 consecutive patients were analyzed with ROSE: bronchoscopy with transbronchial needle aspiration (TBNA) had been performed in 72 patients and with endobronchial ultrasound (EBUS)-TBNA in 201. Cytomorphological features were correlated with the final diagnosis and diagnostic accuracy was measured. Analysis of the different cytomorphological parameters showed that the best sensitivity and specificity were obtained for adenocarcinoma by combining the presence of nucleoli and small/medium cell clusters, and for squamous cell carcinoma by considering the presence of necrosis ≥50% and large cell clusters. For small cell carcinoma, the best diagnostic accuracy was obtained by combining moderate necrosis (<50%) and the presence of single cells. Overall accuracy ranged from 90% to 97%. We showed that it was possible to establish the histotype of the most frequent lung cancers during ROSE using only a few easily identifiable cytomorphological parameters. An accurate diagnosis during ROSE could help endoscopists to decide how many tumor samples must be taken, *e.g*.a higher number of samples is needed for the biomolecular characterization of adenocarcinoma, whereas one sample may be sufficient for squamous cell carcinoma.

## INTRODUCTION

Lung cancer is the most common cause of death from cancer worldwide and only 30-40% of all lung cancers are operable [1, 2]. The only available diagnostic material often comes from biopsy or from cytological samples obtained by fine needle aspiration (FNA). In the majority of cases, the final diagnosis is based on cytological evaluation and it is thus important to use the same biological material for molecular analysis [2–4].

Fine needle aspiration can be performed under radiological (conventional transbronchial needle aspiration - TBNA) or ultrasound guidance (endobronchial ultrasound (EBUS)-TBNA) [5, 6]. The TBNA procedure is performed using a flexible fiberoptic bronchoscope to obtain tissue or fluid samples from the lungs and surrounding lymph nodes without conventional surgery [7]. EBUS is an invasive but highly effective procedure used to diagnose lung cancer, infections and other diseases causing enlarged lymph nodes in the chest [8].

The percentage of tumors diagnosed by cytological evaluation has increased since the advent of EBUS-TBNA [8]. EBUS permits physicians to perform FNA on paratracheal and/or mediastinal lesions [9, 10]. In fact, metastases in homo- and/or controlateral peribronchial and mediastinal lymph nodes can be more easily diagnosed and staged using this technique [1, 9–11]. For inoperable tumors, cytology is the only valid tool for both diagnosis and biomolecular characterization [2–4].

Rapid on-site evaluation (ROSE) is a useful technique to rapidly assess the adequacy of biological material obtained by FNA [12–14] in terms of its cellularity. In eliminating unnecessary sampling, it also helps to reduce costs [15, 16]. Given that ROSE is a widely used technique, different evaluation criteria have been proposed to determine specimen suitability for samples obtained by EBUS-TBNA, *e.g*. amount of lymphoid elements, presence of polluting bronchial cells, number of pigmented macrophages, amount of necrosis and presence of mucus and blood [15–18]. Jeffus et al. performed a comparison between Minnesota and New York criteria to establish the adequacy of EBUS-TBNA specimens [15], while Idowu and Powers analyzed the cytomorphological characteristics of primary lung cancers. In particular, typical cytomorphological features of adenocarcinoma include cellular clusters or acinar (glandular) arrangements, large vacuolated cells, high nuclear/cytoplasm (N/C) ratio, prominent nucleoli and finely granular chromatin [19]. From a cytomorphological point of view, well differentiated squamous cell carcinoma typically appears as individual cells or cohesive sheets of tumor cells, depending on the method of specimen procurement, with well-defined cell borders and dense cytoplasm. Small cell carcinomas typically consist of a biphasic population of viable and degenerating cells arranged in single or loose clusters with nuclear molding, individual cell necrosis and tumor diathesis. The viable tumor cells have a high N/C ratio, smooth nuclear membranes, finely granular evenly dispersed chromatin and inconspicuous or absent nucleoli [19]. The morphological parameters used to determine sample adequacy for ROSE are the same as those of conventional cytology. However, they are time-consuming, require the skills of an expert, and some are only visible with specific stains (*e.g*. chromatin with Papanicolau). There are still no easily detectable and reproducible cytomorphological features that can be used during ROSE to identify tumor histotype and facilitate the number of tissue samples to be taken, especially in adenocarcinoma where more samples are needed for biomolecular characterization. We thus decided to focus our attention on this issue.

The cytomorphological features recorded on site were retrospectively compared to the final diagnosis made on the formalin-fixed and paraffin-embedded (FFPE) material of the cytological specimen and further confirmed on the tumor tissue obtained by biopsy during broncoscopy and/or on resected tumor tissue from operable lesions. We also planned to evaluate the sensitivity and specificity of the identified criteria.

## RESULTS

Histological diagnosis showed 131 (48%) lung tumors and 22 (8%) non-lung tumors. One hundred and twenty (44%) samples were derived from non-tumor material (Table 1). Non-lung tumors were diagnosed exclusively by EBUS-TBNA. Samples obtained by TBNA were derived from lung tumors or normal lung tissue. Ninety (45%) of the samples obtained by EBUS-TBNA were composed of reactive lymph node material (Table 1). 90% of the tumor samples obtained by EBUS-TBNA were from non-operable tumors that were metastatic at diagnosis.

**Table 1:** Distribution of FNA specimen types according to histological diagnosis

	No. (%)	Diagnosis	No. (%)	Type of FNA	
				EBUS-TBNA	TBNA
				No. 201	No. 72
Lung tumors	131 (48)	Adenocarcinoma	80 (61)	46	34
		Squamous cell carcinoma	23 (18)	9	14
		Small cell carcinoma	19 (15)	15	4
		Adenosquamous carcinoma	4 (3)	1	3
		Neuroendocrine large cell carcinoma	3 (2)	1	2
		Undifferentiated carcinoma	1 (1)	0	1
		Anaplastic large cell carcinoma	1 (1)	1	0
Non lung tumors	22 (8)	Mesothelioma	1 (5)	1	0
		Thyroid papillary carcinoma	1 (5)	1	0
		Ductal breast carcinoma	2 (9)	2	0
		Oropharingeal squamous carcinoma	1 (5)	1	0
		Thymoma	1 (5)	1	0
		Ovarian carcinoma	1 (5)	1	0
		Prostate adenocarcinoma	2 (9)	2	0
		Kidney adenocarcinoma	3 (14)	3	0
		Colon adenocarcinoma	5 (23)	5	0
		Lymphoma	3 (14)	3	0
		Sarcoma	2 (9)	2	0
Other	120 (44)	Reactive lymph nodes	90 (75)	90	0
		Microgranulomatous lymphadenitis	15 (13)	15	0
		Necrosis	1 (1)	1	0
		Healthy	14 (12)	0	14

FNA = fine-needle aspiration; EBUS-TBNA = endobronchial ultrasound-transbronchial needle aspiration

In contrast, the majority of the peripheral lesions diagnosed by conventional TBNA were operable. Seventy-nine (88%) of the 90 patients with reactive lymph nodes were followed up for 2 years. The initial diagnosis of reactive lymph nodes was confirmed in 74 (94%) cases, while 5 (6%) patients had a different final diagnosis: one adenocarcinoma, 3 sarcoidosis and one tuberculosis infection. Of the 14 specimens defined as non-tumoral by the cytological evaluation of TBNA material, 10 (71%) were subsequently diagnosed as peripheral adenocarcinomas by CT-guided biopsy.

**Figure 1:**
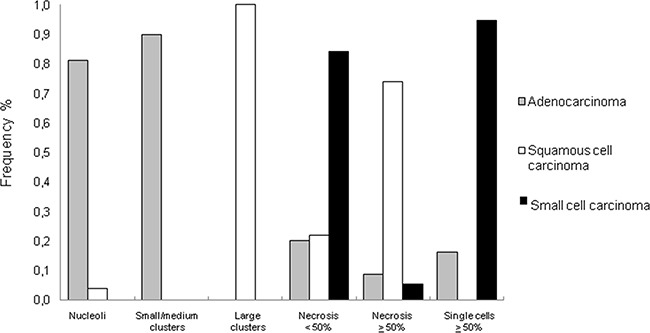
Frequency of cytomorphological features in different lung cancer histotypes

**Figure 2:**
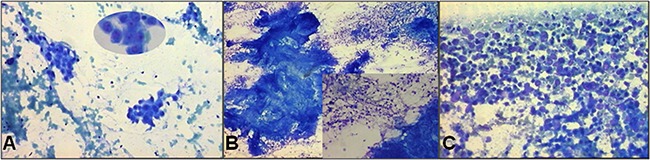
Cytological smears stained with fast quick MGG **A**. A typical histological pattern of adenocarcinoma (10X magnification) with evident nucleoli (visible in the oval field at 20X magnification) and small/medium cell clusters. **B**. A typical histological pattern of squamous cell carcinoma (5X magnification) with large cell clusters and abundant necrosis (visible in the square at 10X magnification). **C**. A typical histological pattern of small cell carcinoma with necrosis and single cells (10X magnification).

**Figure 3:**
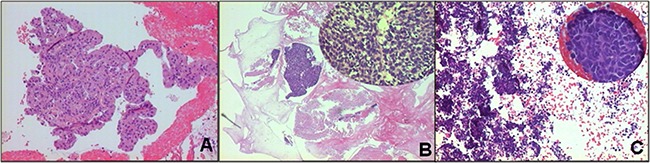
FFPE cytological specimens stained with hematoxylin-eosin for the final diagnosis **A**. Adenocarcinoma histotype (10X magnification). **B**. Squamous cell carcinoma histotype (10X magnification). **C**. Small cell carcinoma histotype (10X magnification).

The distribution of cytomorphological features in relation to tumor histotype is shown in Figure 1. In particular, the presence of nucleoli and small/medium cell clusters was observed in 65 (81%) and 72 (90%) adenocarcinoma histotypes, respectively. The latter characteristic was almost always indicative of the presence of adenocarcinoma (Figure 2A, Figure 3A).

Squamous cell carcinomas were characterized by the presence of large cluster-forming cells and necrosis ≥50%. The presence of large clusters was an exclusive characteristic of this tumor histotype (Figure 2B, Figure 3B). Finally, virtually all (95%) of the small cell carcinomas showed ≥50% single cells (Figure 2C, Figure 3C), while 84% showed moderate necrosis (<50%).

Analysis of the different cytomorphological parameters revealed that the best sensitivity and specificity for adenocarcinoma were obtained by combining the presence of nucleoli and small/medium cell clusters. For squamous cell carcinoma, the presence of necrosis ≥50% and large clusters were considered as the best criteria for an accurate histological diagnosis, with an overall accuracy of 97% (*P*<0.001) determined by the chi-square test. Moreover, the best overall diagnostic accuracy of 94% (*P*<0.001) for small cell carcinoma was obtained by combining the characteristics of moderate necrosis (<50%) and single cells (Table 2).

**Table 2:** Diagnostic accuracy of cytomorphological features in relation to tumor histotype

Histotype	Cytomorphological features	Sensitivity %	Specificity %	Overall Accuracy %	*p*
Adenocarcinoma	Nucleoli and small-medium clusters	73	98	90	<0.001
Squamous cell carcinoma	High necrosis and large clusters	74	99	97	<0.001
Small cell carcinoma	Moderate necrosis and single cells	79	96	94	<0.001

## DISCUSSION

The physiology and anatomy of the lung makes it difficult to obtain biological material from nodular lesions by biopsy and/or needle aspiration [20]. FNA is a well tolerated diagnostic method that permits diagnostic material to be removed from a solid lesion. Although an invasive procedure, it is almost always risk-free. The majority of peripheral lung nodules and peribronchial/mediastinal lymph nodes can only be evaluated by TBNA and EBUS-TBNA, respectively [9–11]. Thus, adequate cytological material in terms of both quality and quantity is essential for diagnosis and for providing biological information that can be used to tailor therapy. Several studies have already demonstrated that cytological material is suitable for molecular analysis [2–4].

Some authors have reported diagnostic difficulties and pitfalls of ROSE for material obtained by EBUS-TBNA [21–23]. In their paper, “The petals and thorns of ROSE (rapid on-site evaluation)”, da Cunha Santos et al. reported an improvement in sensitivity and the need for less additional sampling (with a consequently lower risk for the patient), but also highlighted the importance of having an experienced on-site cytopathologist [21]. Moreover, several authors have also evaluated the potential correlation between ROSE and the final histological diagnosis [24–27]. However, there are still no “user-friendly” criteria to adopt during ROSE to histologically classify cytological specimens and a multidisciplinary approach involving a pulmonologist, radiologist and cytopathologist is required [26, 27].

Although we were not able to classify all lung cancer types, we demonstrated that it was possible to establish the histological type of the most frequent lung tumors during ROSE using a few easily identifiable cytomorphological features. Another weak point of our work was its retrospective nature and the low number of patients with squamous cell carcinoma or small cell lung cancer. However, the numbers reflect the normal distribution and prevalence of these tumor histotypes in a population of patients with lung cancer.

The adenocarcinoma histotype was characterized by the presence of one or more nucleoli and small/medium cell clusters, both of which showed high specificity and sensitivity. Necrosis was observed in some cases but did not exceed 50% of the cytological samples. The presence of abundant necrosis was an indication of metastasis from adenocarcinoma of the colon, breast or prostate. Large clusters were observed in almost all squamous cell carcinomas, often characterized by abundant necrosis (≥50%). In contrast, moderate necrosis (<50%) observed together with the single cell parameter was indicative of a small cell carcinoma histotype.

An accurate diagnosis obtained by ROSE helps to define the number of samples to be taken. In cases of squamous cell or small cell carcinoma, a single sample may be sufficient, whereas multiple samples are needed for adenocarcinoma to permit biomolecular characterization. We demonstrated that a rapid lung cancer diagnosis was feasible during ROSE as it not only enabled us to establish whether the amount of aspirated material was sufficient but also identified the tumor histotype. Given that not all centers have staff trained in cytopathology, the ROSE procedure has also been taught to other professionals, *e.g*. pulmonologists [27, 28]. Our findings could thus be used to train unskilled staff in histotype diagnosis via ROSE as in-depth knowledge of cell morphology is not required.

## MATERIALS AND METHODS

In this retrospective study, cytological specimens were analyzed from 273 consecutive patients who underwent bronchoscopy with TBNA (*n* = 72) or EBUS-TBNA (*n =* 201) at Santa Maria delle Croci Hospital in Ravenna (Italy) between January 2011 and December 2012. The study protocol was reviewed and approved by the institutional ethics committee (Ethics Committee of Area Vasta Romagna, approval no. 611). Written informed consent was obtained from all participants. For each patient, age and sex, site of fine-needle aspiration, original ROSE report (cell block status (yes/no), final diagnosis, and clinical/surgical follow-up (if available) were recorded.

The instrumental analyses were performed by the pulmonologist in the operating theater in the presence of the pathologist, nurses and the anesthesiologist. Lung lesions were evaluated by video-fiberscope (PENTAX EB1570K). TBNA biological specimens were obtained by Wang (21-22 gauge) transbronchial cytology needles under fluoroscopic guidance. Patients undergoing TBNA were mildly sedated with midazolam (Accord Healthcare Limited, Middlesex, UK) and local lidocaine (Bioindustria, L.I.M, Novi Ligure, Italy).

Mediastinal/hilar lymph node lesions and peribronchial lesions were analyzed by echo-endoscopy (EBUS PENTAX, Miyagi Factory HOYA Corporation, Japan). Lymph node stations 7, R4/L4, R2/L2 and 10 were evaluated by EBUS, while stations 5, 8 and 9 were assessed by endoscopic ultrasonography.

Twenty-two-gauge fine needles (Medi-Globe GmbH, Germany) were used in patients under general anesthesia by laryngeal mask airway intubation. A part of the first cytological sample was smeared by the pathologist on a *positively charged slide for analysis with ROSE*. The remaining material was fixed in 10% formalin and embedded in paraffin to obtain a tissue block. The quantity and quality of the samples were established for each patient by ROSE.

All cytological smears were immediately fixed using 80% alcohol and stained with Fast Quick-May-Grunwald Giemsa (MGG) (Diapath Spa, Martinengo, Italy). The entire procedure took 30/40 seconds. The slides were placed directly under the microscope (Axioskop, Carl Zeiss, Gottïngen, Germany) for analysis at 5X and 10X magnification. In the event of insufficient and/or non-diagnostic material, a part of the subsequent sample was used to obtain an adequate cytological smear for ROSE. When ROSE was feasible, at least 2 or 3 samples were taken from the same anatomic site and the material obtained was paraffin-embedded for use in ancillary assays and biomolecular investigations. The final diagnosis was made by 2 pathologists on the FFPE material of all the cytological specimens stained with hematoxylin-eosin using immunohistochemistry (TTF1, P40/P63, CK7, CK5/6, synaptophysin and chromogranin A). The diagnosis was further confirmed on the tumor tissue obtained by biopsy during broncoscopy and/or on resected tumor tissue in operable lesions.

In addition to evaluating the quality of the cytological samples, 3 easily identifiable cytomorphological features were analyzed on site by the pathologist and the information was recorded in a specific form for each patient:

a) Presence of one or more nucleoli.

b) Cellular organization (cells arranged in small/medium or large clusters or the presence of individual non cluster-forming cells). Groups of cells visualized in a microscope field at 10X magnification were defined as small/medium clusters. In contrast, cells not visualized in a field at 10X magnification were classified as large clusters.

c) Amount of necrosis (< 50% or ≥50%) in the entire cytological sample.

These parameters were retrospectively analyzed and compared with the final diagnosis made on the FFPE sample.

### Statistical analyses

The relation between cytomorphological features and tumor histotype was assessed by the chi-square test and corrected with Yates correction [29]. On the basis of the distribution of the different characteristics, sensitivity and specificity were determined by calculating the true-positive (sensitivity) and false-positive (1-specificity) rates. For all tests, a 2-sided *P* = 0.05 was regarded as significant.

## Abbreviations

FNA: fine needle aspiration
ROSE: rapid on site evaluation
TBNA: transbronchial needle aspiration
EBUS: endobronchial ultrasound
FFPE: formalin-fixed paraffin-embedded
IHC: immunohistochemistry
MGG: May-Grunwald Giemsa
TTF1: thyroid transcription factor 1
CK: cytokeratin
CT: computed tomography
